# Drug-loaded hyaluronic acid hydrogel as a sustained-release regimen with dual effects in early intervention of tendinopathy

**DOI:** 10.1038/s41598-019-41410-y

**Published:** 2019-03-18

**Authors:** Ming-Yen Hsiao, An-Ci Lin, Wei-Hao Liao, Tyng-Guey Wang, Chia-Hsien Hsu, Wen-Shiang Chen, Feng-Huei Lin

**Affiliations:** 1 Department of Biomedical Engineering, National Taiwan University, Taipei, Taiwan; 2 Departments of Physical Medicine and Rehabilitation, National Taiwan University Hospital, College of Medicine, National Taiwan University, Taipei, Taiwan; 3 Institute of Biomedical Engineering and Nanomedicine, National Health Research Institutes, Maioli, Taiwan; 4 Director, Institute of Biomedical Engineering and Nanomedicine, National Health Research Institutes, Maioli, Taiwan

## Abstract

Resulting from accumulative microtrauma, impaired healing and oxidative stress, tendinopathy is a debilitating and relentlessly deteriorating disease that greatly affects daily function and quality of life. Current therapy usually provides symptomatic relief only. Sufferers undergo repetitive and protracted treatment courses that rarely alter the disease process. We aim to develop a sustained-release regimen with an intrinsic therapeutic effect in tendinopathy treatment, using oxidised hyaluronic acid/adipic acid dihydrazide hydrogel (HA hydrogel) as both the drug carrier and a mitigating agent of symptoms. We show that HA hydrogel can mitigate tendinopathy changes both *in vitro* (mechanically induced tendinopathy model) and *in vivo* (collagenase-induced tendinopathy model). A potent anti-oxidative (pigallocatechin gallate) incorporated into HA hydrogel conferred an additional protective effect in both models. The results indicate that when administered early, combined medications targeting different pathogenesis pathways can resolve tendinopathy. Although facilitating the healing process and mitigating oxidative stress are promising therapeutic strategies, the most effective regimen for tendinopathy treatment has to be determined yet. The established experimental model and drug carrier system provide a platform for exploring new therapeutics against this debilitating disease.

## Introduction

Tendinopathy is a prevalent and debilitating musculoskeletal disorder, estimated to account for over 30% of overuse injury cases in musculoskeletal systems^1–3^. The natural course of the disease is variable. In some patients the tendinopathic process remains relatively stationary, while in some population it relentlessly deteriorates, ultimately leading to tendon tear. However, tendinopathy therapy has progressed little in the last several decades. Most current treatments provide symptomatic relief only, without altering the disease course^4^. Among the few possible disease-modifying therapies is regenerative therapy. Although promising results have been reported in certain populations, controversy exists regarding the exact mechanisms and definite efficacy^5,6^. While the standard protocol is to be developed, it usually requires repetitive injections and protracted treatment courses over weeks to months.

**Figure 1:**
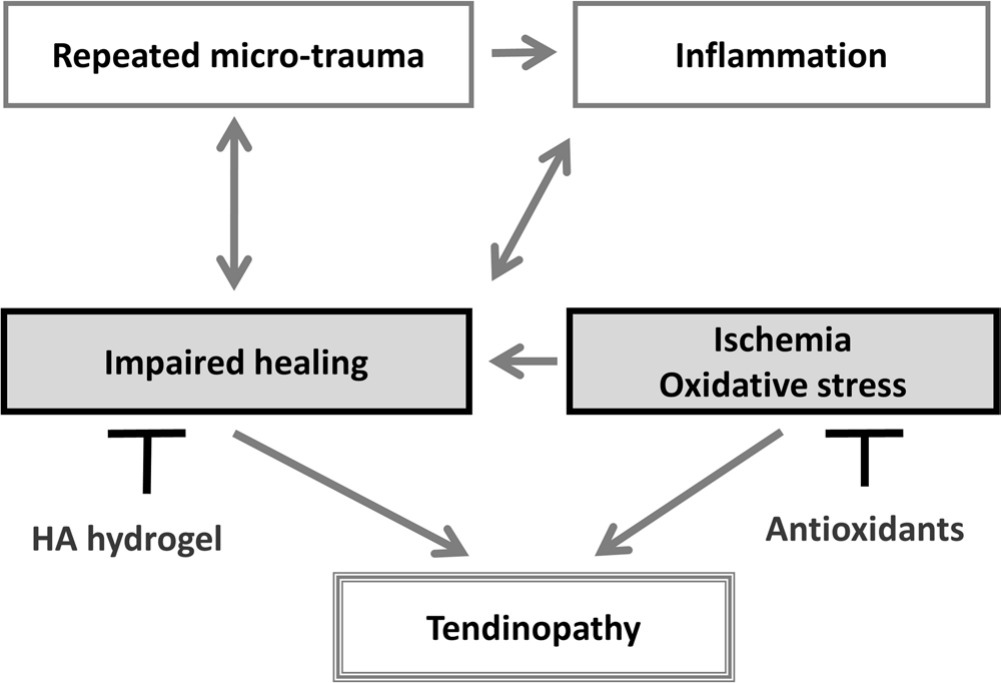
Proposed pathogenesis of tendinopathy and possible early interventions using HA hydrogel as a drug carrier loaded with anti-oxidative medications, and a therapy facilitating healing process.

The most accepted theory of tendinopathy aetiology is mechanical overloading, repetitive injuries and inflammation causing accumulated microtraumas and tissue destruction exceeding the healing capacity of the tendon^1,2,7,8^, which is often accompanied by scar formation, fibrosis and adhesion, which reduces mechanical strength and exerts excessive shear force during tendon motion. This increases the risk of secondary injury^1,3,9^. The healing process also induces an inflammatory response, generating reactive oxygen species. Additionally, ischaemia increases the oxidative stress, predisposing the tendon to injury and impairing the healing process^1,2,10,11^ (see Fig. 1).

Successful treatment of tendinopathy relies on early interruption of the degenerative process, which becomes irreversible once fully developed. As anti-inflammation medication has limited effect in chronic tendinopathy, we propose facilitating the healing process and mitigating the oxidative stress (Fig. 1). In clinical applications, a single sustained-release injection dosage with a prolonged therapeutic effect (over several weeks) is highly desirable.

Hyaluronic acid (HA) is a well-known anti-fibrotic and anti-inflammatory agent that reduces scar formation and adhesion during the early stages of tissue healing^12–16^. It has prevented post-operation tendon adhesion in both animal and human studies^17^ and has been used in many degenerative conditions such as osteoarthritis^14,16^. Direct tendon injection of HA maintains the architecture, reduces microtearing and decreases apoptosis in rat tendinopathy models^18,19^. A biocompatible and thermosensitive material comprising oxidised hyaluronic acid/adipic acid dihydrazide hydrogel (HA hydrogel) forms a gel within three minutes of *in vivo* injection, and retains its gel-like state for up to five weeks *in vivo* condition^20,21^. Utilising these properties, we deploy HA hydrogel as a sustained-released drug carrier in an early tendinopathy intervention. Simultaneously, we exploit the possible therapeutic effect of HA hydrogel per se on the healing process. As a proof-of-concept, we combine HA hydrogel with epigallocatechin gallate (EGCG), a natural polyphenol with potent anti-oxidative and anti-inflammatory properties^22,23^, and evaluate its effect in a cyclic uniaxial stretching *in vitro* model and a collagenase-induced *in vivo* rat tendinopathy model.

## Results

### Cell viability assay and drug release profile of EGCG

**Figure 2:**
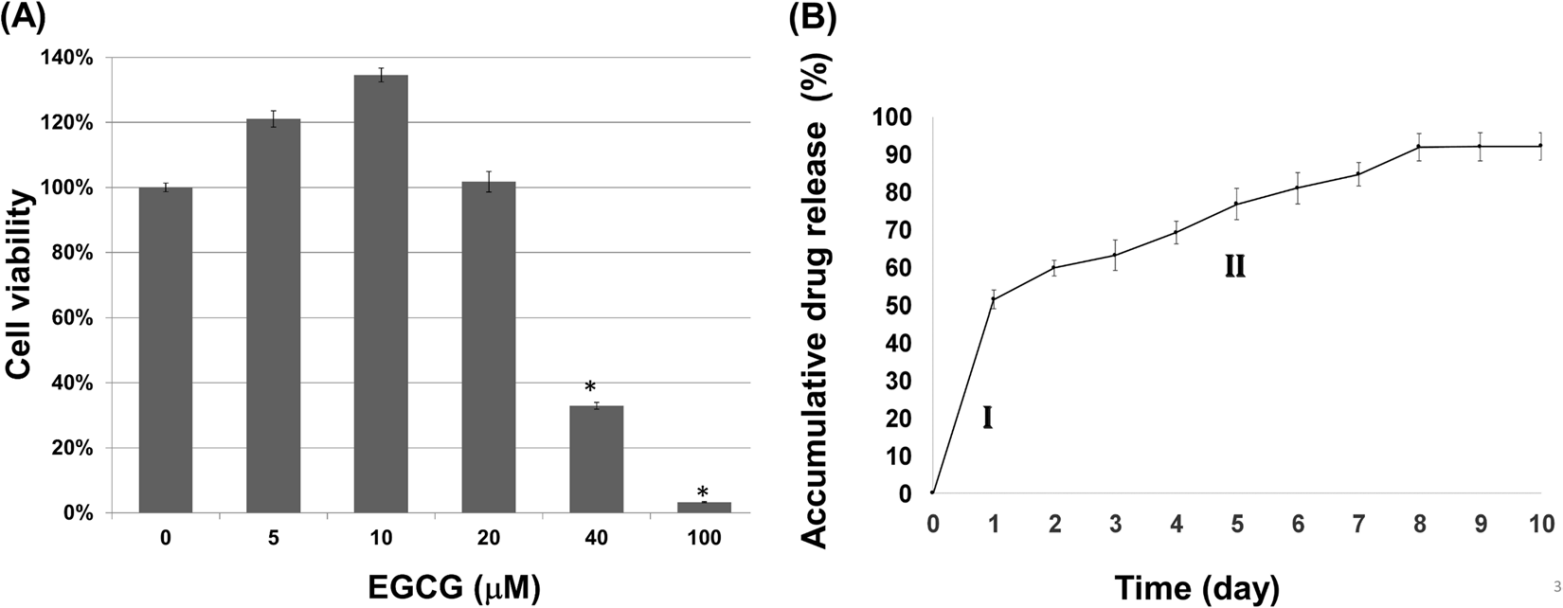
Cell viability assay and drug release profile. (**A**) WST-1 assay showing cell viability (percentage) of TDCs at different EGCG concentrations. Data are represented as means ± SDs (*p < 0.05). (**B**) Cumulative release profile of EGCG-loaded hydrogel showing the two-phase release of EGCG: an initial burst (I) and steady release (II).

The WST-1 assay of the tendon-derived cells (TDCs) revealed no significant toxicity of EGCG at concentrations of 5, 10, and 20 μM (Fig. 2). In subsequent cell-culture experiments, the EGCG concentration was set to 10 μM, following a previous *in vitro* study on the protective effect of EGCG on intervertebral disc cells^24^. The cumulative release profile of the EGCG-loaded hydrogel showed a two-phase release of EGCG, an initial burst of 51.5% during the first 24 h, followed by a steady release of 40.8% over 1–10 days (Fig. 2). Up to 92.2% of the EGCG was released over 10 days, confirming a therapeutic interval after *in vivo* injection. In this study, the HA hydrogel regiment was cross-linked with 8% adipic acid dihydrazide (ADH) (w/v), which is biocompatible with a degradation period of five weeks or less under *in vivo* conditions^20^.

### Effect of EGCG-loaded hydrogel on the gene expressions of tendon-derived cells (TDCs) under cyclic uniaxial stretching

**Figure 3:**
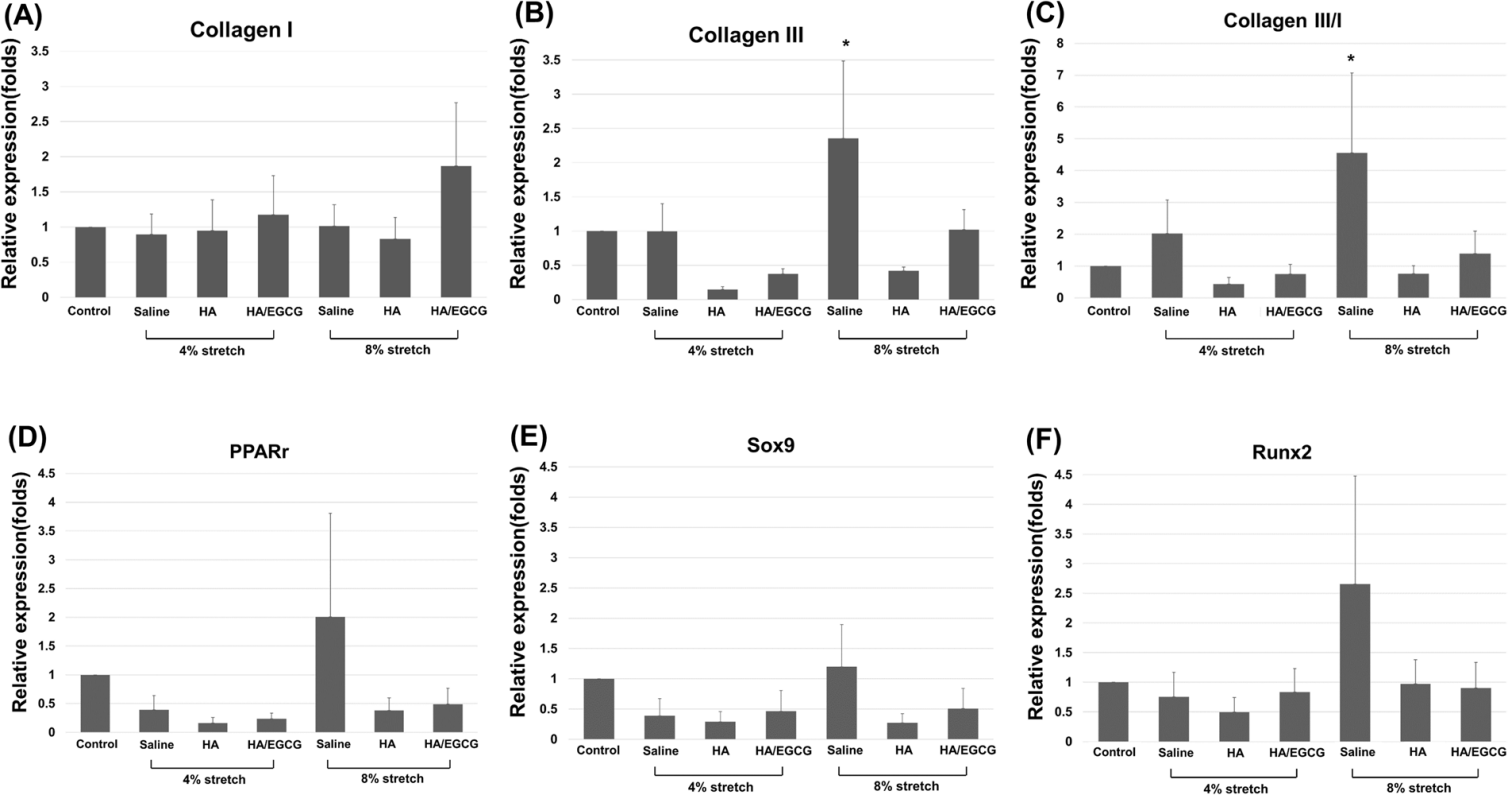
Effect of HA hydrogel and EGCG-loaded hydrogel (10 μM EGCG) on the gene expression of TDCs (*n* = 4) subjected to cyclic stretching under 4% or 8% strain. Medications were added to the upper layer of the tranwell. Gene expression ratio of (**A**) collagen I, (**B**) collagen III, (**C**) collagen III/ collagen I, (**D**) *Pparg* (adipogenic), (**E**) *Sox9*(chondrogenic) and (**F**) *Runx2* (osteogenic). All ratios were detected by qPCR. Data are expressed as mean ± SD (*p < 0.05) fold-changes of the expression level relative to the control (static culture). HA, HA hydrogel; HA/EGCG, EGCG-loaded hydrogel.

The 4% and 8% strain of cyclic uniaxial stretching were set according to previous *in vitro* studies^25,26^.Three days after cyclic stretching, qPCR showed that the expression ratio of collagen III/collagen I genes in the TDCs (a miscellaneous collection of tendon-derived stem cells, tenoblasts, tenocytes and fibroblasts) increased 4-fold in the 8% strain group treated with saline (*p* < 0.05), but not in 4% group. The increased expression ratio in the 8% strain group was suppressed by addition of HA hydrogel or EGCG-loaded hydrogel (*p* < 0.05) (Fig. 3). The gene expressions of the peroxisome proliferator activated receptor gamma (*Pparg*, adipogenic), *Sox9* (chondrogenic) and Runt-related transcription factor 2 (*Runx2*, osteogenic) genes showed similar trends but were not statistically significant (Fig. 3).

### Effect of in vivo injection of EGCG-loaded hydrogel on collagenase-induced tendinopathy

#### Histology

**Figure 4:**
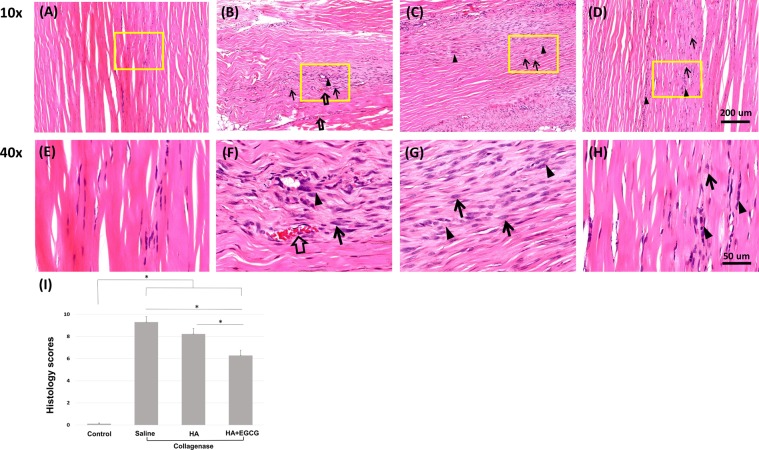
Representative micrographs of H&E-stained Achilles tendon specimens (*n* = 12) 14 days after collagenase injection. (**A**,**E**) normal tendons (control group, injected with saline only), and collagenase groups injected with (**B**,**F**) saline, (**C**,**G**) HA hydrogel and (**D**,**H**) EGCG-loaded hydrogel. Arrows, rounded nucleus of tenocytes; arrowheads, focal hypercellularity; blank arrows, increased vascularity. (**I**) Histological evaluation scores of the H&E-stained tendon specimens 14 days after collagenase injection. Data are presented as means ± SDs (*p < 0.05). (**A–D**), 10 × H&E-stained; (**E**–**H**), 40 × H&E-stained from yellow box in (**A–D**), respectively.

On day 14, the haematoxylin and eosin (H&E) stains of tendon specimens from all groups injected with collagenase showed marked disorganisation and thinning of the collagen fibres, rounded tenocyte nuclei, focal hypercellularity and higher vascularity than the control group (injected with saline rather than collagenase). Vascularity was noted by luminal structures and red blood cells within. In the group treated with EGCG-loaded hydrogel one day after collagenase injection, the collagen fibre structures were more organised and thicker than in the saline- and HA hydrogel-treated groups, with less cellularity and vascularity. The collagen fibres in the control group were regularly aligned and interspersed with tenocytes with spindle-shaped nuclei. Cellularity and vascularity were rare (Fig. 4).

The semi-quantitative histological evaluation scores^27^ were lower in the group treated with EGCG-loaded hydrogel than in the HA hydrogel- and saline-treated groups (*p* < 0.05), but all groups scored significantly higher than the control group (Fig. 4).

### Effect of EGCG-loaded hydrogel in vivo injection on collagenase-induced tendinopathy Gene expression

**Figure 5:**
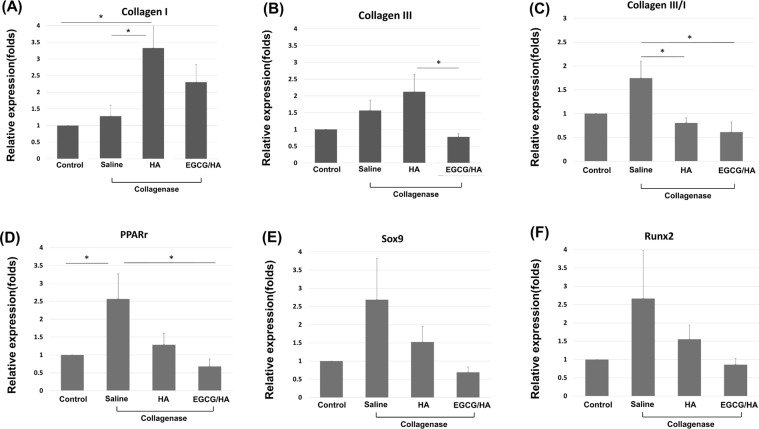
Gene expressions of Achilles tendon (*n* = 12) 14 days after collagenase injection. Gene expression ratio of (**A**) collagen I, (**B**) collagen III, (**C**) collagen III/collagen I ratio, (**D**) *Pparg* (adipogenic), (**E**) *Sox9* (chondrogenic) and (**F**) *Runx2* (osteogenic). The control group was injected with saline instead of collagenase. Data are expressed as mean ± SD (*p < 0.05) fold-changes of expression level relative to the control. HA, HA hydrogel; HA/EGCG, EGCG-loaded hydrogel.

The expression of *Pparg* was higher in the collagenase-induced tendinopathy sample treated with saline injection than in the control group (*p* < 0.05), but the expressions of the type I and type III collagen genes, *Runx2* and *Sox9* were similar between the saline-treated and control groups. The elevated *Pparg* in collagenase-induced tendinopathy was suppressed by EGCG-loaded hydrogel (*p* < 0.05). Moreover, the expression ratio of type III/type I collagen in both the EGCG-loaded hydrogel and HA hydrogel groups of collagenase-induced tendinopathy was lower than that in the saline-treated sample (*p* < 0.05). The expression of type III collagen in the EGCG-loaded hydrogel group was lower than that in the HA hydrogel group (*p* < 0.05). HA hydrogel did not affect the expression of type III collagen but increased the expression of type I collagen when compared with the saline-treated sample (*p* < 0.05). The expressions of the *Runx-2* and *Sox9* showed similar trends without statistical significance (Fig. 5).

## Discussion

Our study confirmed the feasibility of HA hydrogel as a drug carrier that can be loaded with potential medications, achieving a sustained-release drug delivery with possible intrinsic therapeutic effects. The HA hydrogel mitigated the tendinopathy changes in both *in vitro* mechanically induced and *in vivo* collagenase-induced tendinopathy models. As a proof-of- concept, the potent anti-oxidative EGCG was incorporated into the HA hydrogel. Although further refinement is necessary, the regimen showed an additional protective effect in the histological investigations, and further diminished the expressions of tendinopathy-associated genes *in vivo*. Therefore, early treatment with combined medications targeting different pathogenesis pathways might arrest the degenerative disease. Facilitating the healing process and mitigating the oxidative stress is particularly a promising therapeutic strategy, although the most effective regimen against tendinopathy has to be determined yet.

HA hydrogel, formed by oxidised and crossed-linked HA, is biocompatible and biodegrades after a long post-injection residence time^20,28^. Therefore, it is a promising drug carrier candidate. Our study implies that HA hydrogel and HA confer similar protective effects against tendinopathy. Besides possessing intrinsic anti-fibrotic and anti-inflammatory properties^12–16^, HA protects tendons against the detrimental effects of overuse or detraining^29^. Previously, repeated HA injections effectively relieved pain and decreased the microtearing and apoptosis in the patellar tendons of a rat tendinopathy model induced by treadmill running^18^. Additionally, repeated HA injection helped to maintain the structure and morphology of the patellar tendons in detrained rats^19^. Our study reconfirms the beneficial effect of HA injection in tendinopathy. Furthermore, a single injection of drug-loaded HA hydrogel is a promising sustained-release regimen in the collagenase-induced tendinopathy model. Loading with other potential disease-modifying medications might synergise the therapeutic effect of HA hydrogel.

Owing to its potent anti-oxidative properties^22,23^, EGCG confers anti-carcinogenesis, anti-ageing and cardioprotective properties^22,23,30^. Accumulated evidence has indicated an important role for oxidative stress in tendinopathy^31–33^, and anti-oxidants are cytoprotective for tenofibroblasts^34^. EGCG also inhibited the cell death related to reactive oxygen species in a human intervertebral disc cell model^24^. Thus, we hypothesised that EGCG can mitigate the ischaemia/oxidative injury associated with repetitive microtrauma and inflammation in tendinopathy, having a similar degenerative nature. We provide the first demonstration that EGCG incorporated into HA hydrogel is a potentially effective injection regimen in a tendinopathy model. Although the positive effect possibly results from the anti-oxidative properties of EGCG, it requires further investigation.

In this study, both HA hydrogel and the EGCG-loaded hydrogel suppressed the increasing expression ratio of collagen III/I in the tendinopathy group. Consistent with this finding, HA regulates the fibrosis process and scar formation^12–16^. An increased collagen III/collage I ratio in the extracellular matrix (ECM) is a prominent histological change in tendinopathy^10,11,35^. Type III collagen, the major component in early scar tissue, is more irregularly organized and mechanically weaker than type I collagen. The overloaded condition was simulated under similar excessive axial strain in previous *in vitro* studies, in which the non-tenocyte lineage differentiation began after eight hours of cyclic loading^25,26,36^. Our study tested the mechanical loading effect over three days, and obtained comparable gene expressions of collagen III/I.

The expressions of *Pparg* (adipogenic), *Sox9* (chondrogenic) and *Runx2* (osteogenic) were not increased by mechanical loading in our *in vitro* model, and *Pparg* alone increased in our *in vivo* model. Notably, the expressions of these three genes showed a similar trend but with substantial variability. To observe a significant increase in the non-tenocyte lineage genes, the induction duration in both models might need to be lengthened. In fact, tissue metaplasia such as chrondro-osteogenic differentiation are commonly observed in late-stage tendinopathy^37–39^. Whether the early (<8 h) increases in these genes after mechanical loading, as observed in previous *in vitro* studies, reflect the acute injury response requires further elucidation.

This study has several limitations. First, the study assessed the feasibility of drug-loaded HA hydrogel as a dual-effect therapy and the applicability of its *in vivo* injection, without exploring the exact mechanism of HA and EGCG on tendon healing. Furthermore, we should identify the most effective medication and optimise the regimen of the loaded medication and HA hydrogel to extend the sustained-release time. Second, the collagenase-induced model better clarifies the healing response after an acute injury than the degeneration process. The histological and gene expression outcomes were examined at two weeks after the collagenase injection, whereas chronic tendinopathy develops over a much longer period. In future studies, weekly repeated collagenase injections and a longer follow-up duration might better simulate the chronic tendinopathy condition, possibly revealing prominent changes of ECM composition and tissue metaplasia. Other pharmacologically-induced animal model of tendinopathy should also be considered. For example, prostaglandin E1 tendon injection induces adhesion, intra-tendinous degeneration and paratenon fibrosis, which may serve as an useful model for future studies^40^. Third, the cells in *in vitro* studies are mechanically loaded without interaction with sufficient ECM. As this condition neglect the actual physiological conditions, the gene expressions might only assay the cellular response to mechanical overloading. Fourth, because the cells proliferate and must be subcultured every 2–3 days, the gene expression was measured on third day. Simulating the long-term degeneration condition *in vitro* is a challenging task, but might be achieved by *ex-vivo* tendon culture in future. Finally, to optimise the proposed tendinopathy treatment, we might require a comprehensive temporal regulation of multiple pathways other than the healing process and oxidative stress. This might necessitate multiple models representing the different stages and conditions of the disease.

## Conclusions and Perspectives

In the present proof-of-concept study, HA hydrogel exhibited sustained-release properties as a drug carrier that can be loaded with potential disease-modifying medications, possibly boosting the intrinsic therapeutic effect. The regimen opens a new window of tendinopathy treatment, in which the relentlessly degenerative process of tendinopathy is arrested at an early stage by inducing multiple pathways, including the healing process and oxidative stress mitigation. The established experimental model and drug carrier system also provide a platform for future exploration of new tendinopathy treatments.

## Materials and Methods

This study followed the National Institutes of Health guide for the care and use of laboratory animals (NIH Publication No. 8023, revised in 1978). All the experiments accorded with the Institution Guidelines and were approved under the Affidavit of Approval of Animal Use Protocol, College of Medicine and College of Public Health, National Taiwan University.

### Study protocol

#### Cyclic uniaxial stretching on tendon-derived cells (TDCs)

TDCs were isolated from the Achilles tendons of Sprague–Dawley rats and cultured for 7 days as described in previous protocols^25,26,36^. Briefly, the Achilles tendons of both limbs were harvested from four male Sprague–Dawley rats (aged 4–6 weeks, each weighing approximately 250–300 g) after euthanising with isofluorane (300–600 ml/min). The middle portion of the Achilles tendon was excised, and the adjacent connective tissues were removed. The tendons were minced, digested with type I collagenase (3 mg/ml; Sigma–Aldrich) for 2.5 h, and then passed through a 70 mm BD Falcon™ cell strainer (BD Biosciences, San Diego, CA, USA), yielding a single-cell suspension. The cells were centrifuged at 300 × *g* for 5 min, washed with sterile phosphate-buffered saline (PBS), and resuspended in Dulbecco’s Modified Eagle Medium, 10% foetal bovine serum (FBS), 100 U/ml penicillin and 100 mg/ml streptomycin (all obtained from Sigma–Aldrich). The isolated cells were cultured at 37 °C with 5% CO_2_ at a cell density of 10^4^ cells/cm^2^. After culturing for 24 hours, the non-adherent cells were removed by washing with PBS. On day 7, the cells were trypsinised and mixed in passage 0 (P0). Cells from passages 1 to 5 (P1–P5) were used in all the experiments as described by previous studies^25,26,36^. The potential of multi-differentiation was sustained up to P6^25,26,36^. The medium was changed every three days.

**Figure 6:**
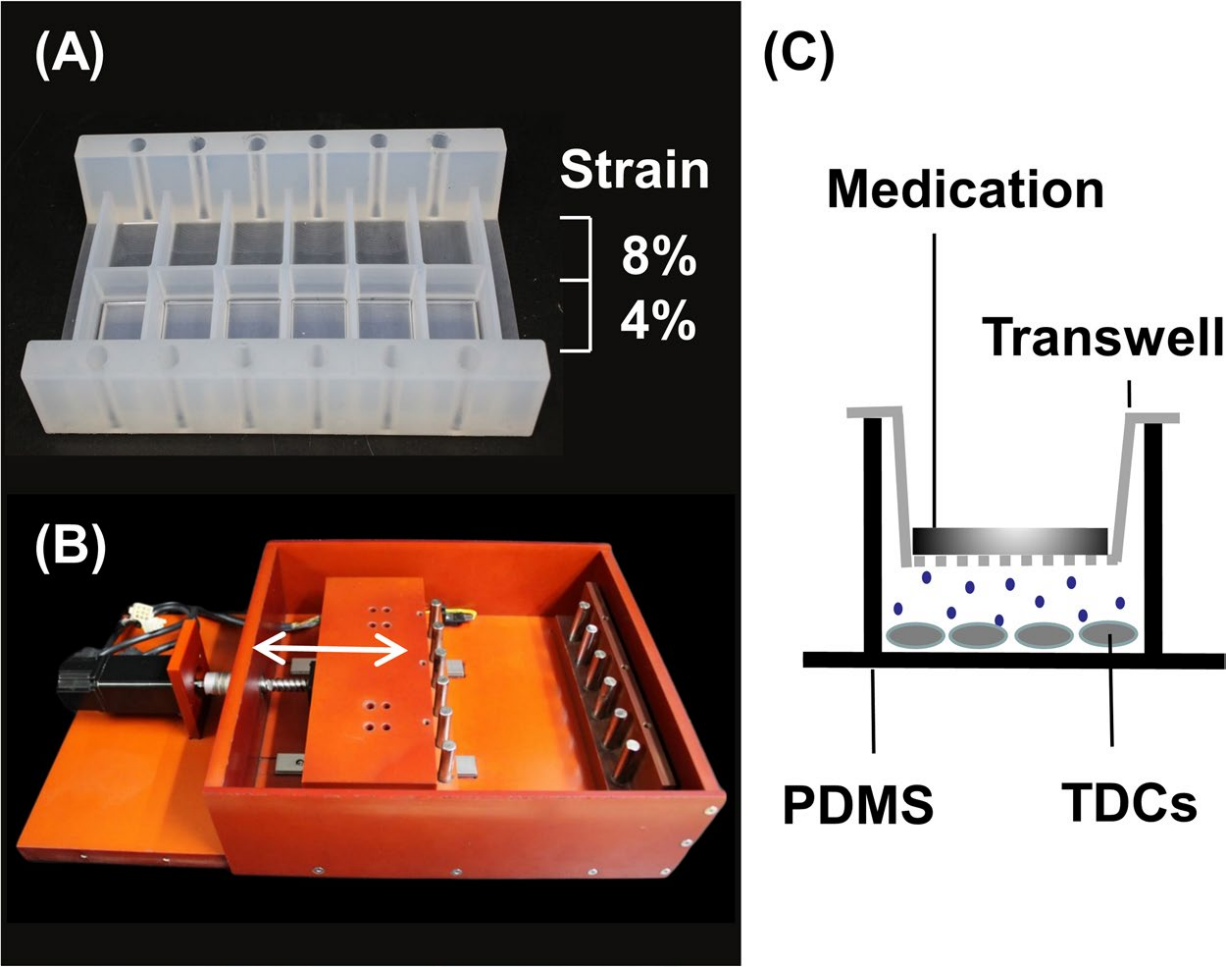
Designed bioreactor with the modified transwell culture system. (**A**) Elastic culture plate made of polydimethylsiloxane (PDMS). Under uniaxial stretching, the strain ratio of the two rows was 1:2. (**B**) Mobile supporting frame operated by a servomotor, which generates cyclic uniaxial stretching. (**C**) The modified transwell culture system. The HA hydrogel and EGCG-loaded hydrogel were placed above the permeable membrane of the transwell, while the TDCs were cultured below the membrane on the PDMS plate. Arrow: stretching direction.

We created a novel bioreactor that simultaneously exerts different uniaxial tensile strains on the same culture plate. The culture plates were specially designed with an elastic property and were constructed from polydimethylsiloxane (PDMS). Each culture plate was partitioned into two rows of chambers with bottoms of different thicknesses. Uniaxial mechanical stretching imparted a strain ratio of 1:2 between the rows (Fig. 6). The uniaxial stretching was cycled in the bioreactor, powered by a servomotor with adjustable displacement and frequency settings.

The isolated TDCs were seeded on a self-designed culture plate topped with a modified transwell (Fig. 1) and cultured for 48 hours. The seeding density was 10^4^ cells/cm2. After replacing the growth medium, the culture plate was placed in the bioreactor and subjected to cyclic uniaxial stretching at 0.5 Hz, 8 h/day, for three days. The strain ratio was varied as 4% and 8%. Cells subjected to either 4% or 8% strain were further divided into 3 groups. Prior to cyclic stretching, groups 1, 2 and 3 were treated with saline, hydrogel alone and epigallocatechin gallate (EGCG) (10 μM; Sigma–Aldrich, St. Louis, MO, USA)–loaded HA hydrogel, respectively. Medications were added to the upper layer of the transwell (Fig. 6). After mechanical loading, the cells were collected, and their RNA was extracted and analysed by quantitative polymerase chain reaction (qPCR). The target genes were types I and III collagen, and non-tenocyte lineage genes encoding PPAR-*r*, Sox-9 and Runx2 are involved in adipogenic, cartilaginous and osteogenic differentiation, respectively^26,36^. All the experiments were performed in triplicate using cells from four rats.

#### Sustained-release regimen in collagenase-induced tendinopathy model

**Figure 7:**
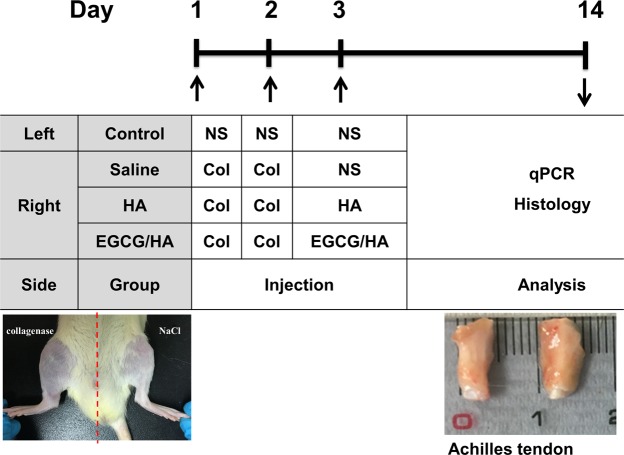
Scheme of injection protocols and outcome measures. Collagenase was injected in Achilles tendons of SD rats (*n* = 12) for two successive days to induce tendinopathy. In the experimental groups, normal saline, HA hydrogel or EGCG-loaded hydrogel were injected on day 3. The control group received a saline injection on days 1, 2 and 3. NS, normal saline; Col, collagenase; HA, HA hydrogel; HA/EGCG, EGCG-loaded hydrogel.

Type I collagenase (0.3 mg dissolved in 30 μl saline, Sigma–Aldrich) was injected at the right side of the Achilles tendons of Sprague–Dawley rats in two successive days, inducing tendinopathy. The injection was performed with a 30 G needle, targeting the central portion of the tendon. On day 3, the right Achilles tendon of each rat was injected with normal saline (group 1), HA (group 2), or epigallocatechin gallate (EGCG)-loaded hydrogel (group 3). Based on previous animal toxicity profiles^21^, which confirmed significant ECGC toxicity above 100 mg/kg, the ECGC dosage was set to 10 mg/kg. The left Achilles tendons of the rats in the control group were injected with saline on days 1, 2 and 3. On day 14, the rats were euthanised by CO_2_ and their Achilles tendons were harvested for histological and qPCR analyses (Fig. 7). All the experiments were performed in triplicate using cells from 12 rats in each group.

### Preparation of HA hydrogel

The HA hydrogel was prepared as described in the previous literature^20^. Briefly, HA sodium salt powder (1 g, Sigma–Aldrich) was dissolved at 1% (w/v) in double-distilled water (100 mL). Sodium periodate solution (NaIO_4_, 2.67%) was gradually added to HA at a molar ratio of 1:1. The oxidation reaction was left to proceed for 24 h at room temperature. The oxidised-HA solution was dialysed for 3 days and then freeze-dried for 3 days. The oxidised-HA was dissolved at 4 °C overnight in PBS (pH 7.4) to 6% (w/v). Meanwhile, ADH was dissolved in PBS to 8% (w/v). Finally, the ADH and oxidised-HA solution were mixed at a ratio of 1:4 to form hydrogel.

To prepare the EGCG-loaded hydrogel, 0.0125 g of EGCG was dissolved in 0.5 ml PBS (20 mg/kg EGCG), and mixed with HA hydrogel (12% w/v oxidised-HA and 6% w/v ADH) at a volume ratio of 1:1. The total EGCG dosage in this mixture was 10 mg/kg and the HA concentration was that of pure HA hydrogel.

### WST-1 Cell viability assay

Spectropmetric quantification with 2-(4-iodophenyl)-3-(4-nitrophenyl)-5-(2,4-disulfophenyl)-2H-tetrazolium monosodium salt (WST-1) reagent (Takara Bio Inc., Japan) was used. TDCs were seeded on the plates at 10^4^ cells/mL and cultured for 48 h. The WST-1 (10 μL) was diluted 1:10 with phosphate buffer, and added to the culture wells. Cells were incubated at 37 °C for an additional 2 h. Cell viability was measured at 450 nm in an ELISA Reader (Infinite M200, Tecan, Austria).

### Drug release profile of EGCG

The EGCG-loaded hydrogel and 3 mL of PBS (pH = 7.4) were added to a 24-well plate and incubated in a shaking bath (37 °C, 50 rpm). From days 1 to 10, the released medium was collected for analysis and replaced immediately with fresh PBS. The EGCG content in the release medium was determined by spectrophotometry (Infinite M200, Tecan, Austria). The cumulative release curve was calculated.

### Quantitative PCR

Quantitative PCR was performed using a Step-One Plus Real-Time PCR System (Applied Biosystems, Lincoln Centre Drive Foster City, CA, USA) according to the manufacturer’s instructions with FastStart Universal SYBR Green Master (Rox) reagent. The primer sequences used in this study were as follows: β – actin: forward 5′-ATCGTGGGCCGCCCTAGGCA-3′, reverse 5′-TGGCCTTAGGGTTCAGAGGGG-3′; Type I collagen: forward 5′-ATCCTGCCGATGTCGCTAT-3′, reverse 5′-CCACAAGCGTGCTGTAGGT-3′; Type III collagen: forward 5′-CTGGTCCTGTTGGTCCATCT-3′, reverse 5′-ACCTTTGTCACCTCGTGGAC-3′; PPAR-*r*: forward 5′-CTGACCCAATGGTTGCTGATTAC-3′, reverse 5′-GGACGCAGGCTCTACTTTGATC-3′; Runx2: forward 5′-CCGATGGGACCGTGGTT-3′, reverse 5′-CAGCAGAGGCATTTCGTAGCT-3′; and Sox-9: forward 5′- CTGAACGAGAGCGAGAAG-3′, reverse 5′-TTCTTCACCGACTTCCTCC-3′. Cytoplasmic RNA was extracted using an RNeasy® Mini Kit and then reverse-transcripted to complementary DNA by the SuperScript III First-Strand Synthesis System.

The PCR conditions were as follows: denaturation at 50 °C for 2 min and 95 °C for 10 min and 40 cycles at 95 °C for 15 s and 58 °C–60 °C for 60 s. ${\rm{\beta }}-{\rm{actin}}$ was used as the internal control. Data were analyzed using the 2^−ΔΔCt^ method and expressed as fold-changes compared with the non-loading group.

### Histology

The Sprague–Dawley rats were euthanised with isofluorane (300–600 ml/min), and the middle portion of their Achilles tendon was excised. The harvested tendon specimens were fixed with 10% formalin, embedded in paraffin and sliced into 7-μm sections. The slides were stained with H&E and observed for fibrillary structure of collagen, cellularity and morphology and vascularity.

Three authors blinded to the experimental groups assessed the semi-quantitative histological evaluation scores. The scoring items were fibre arrangement, fibre structure, angiogenesis, nuclear rounding, inflammation and cell density. Each item was scored as 0 (no tendinopathy), 1, 2, or 3 (most severe tendinopathy)^27^.

### Statistical analysis

The data were presented as mean standard deviation (SD). Comparisons between groups were performed by ANOVA followed by post-hoc Tukey’s tests. All analyzes were done using SPSS version 16.0 software (SPSS Inc., Chicago, IL, USA). Statistical significance was set at p < 0.05.

## Data Availability

The datasets generated during and/or analysed during the current study are available from the corresponding author on reasonable request.
